# How Target-Sequence Enrichment and Sequencing (TEnSeq) Pipelines Have Catalyzed Resistance Gene Cloning in the Wheat-Rust Pathosystem

**DOI:** 10.3389/fpls.2020.00678

**Published:** 2020-05-26

**Authors:** Jianping Zhang, Peng Zhang, Peter Dodds, Evans Lagudah

**Affiliations:** ^1^CSIRO Agriculture & Food, Canberra, ACT, Australia; ^2^Plant Breeding Institute Cobbitty, The University of Sydney, Sydney, NSW, Australia

**Keywords:** wheat rust, TEnSeq, plant immunity, gene cloning, durable resistance

## Abstract

The wheat-rust pathosystem has been well-studied among host–pathogen interactions since last century due to its economic importance. Intensified efforts toward cloning of wheat rust resistance genes commenced in the late 1990s with the first successful isolation published in 2003. Currently, a total of 24 genes have been cloned from wheat that provides resistance to stem rust, leaf rust, and stripe rust. Among them, more than half (15) were cloned over the last 4 years. This rapid cloning of resistance genes from wheat can be largely credited to the development of approaches for reducing the genome complexity as 10 out of the 15 genes cloned recently were achieved by approaches that are summarized as TEnSeq (Target-sequence Enrichment and Sequencing) pipelines in this review. The growing repertoire of cloned rust resistance genes provides new tools to support deployment strategies aimed at achieving durable resistance. This will be supported by the identification of genetic variation in corresponding *Avr* genes from rust pathogens, which has recently begun. Although developed with wheat resistance genes as the primary targets, TEnSeq approaches are also applicable to other classes of genes as well as for other crops with complex genomes.

## Introduction

Wheat crops are afflicted by three major rust diseases, namely stem/black rust, stripe/yellow rust, and leaf rust/brown rust, each caused by a different fungal species in the genus *Puccinia*. Significant yield losses from each of the three diseases have been reported from almost all major wheat-growing regions worldwide. Losses to leaf rust were approximately $350 million between 2000 and 2004 in the United States (Huerta-Espino et al., 2011). In China, leaf rust causes yield losses estimated at 3 million tons annually (Huerta-Espino et al., 2011). Leaf rust was also reported as a severe threat to wheat crops in Mexico and South Asia in the past, but with the utilization of slow rusting resistance genes in some areas like the United States, damage in recent decades has been reduced substantially (Huerta-Espino et al., 2011). Stripe rust epidemics were previously restricted mainly to cooler and humid regions, such as those in Asia and Europe. However, the appearance and spread of aggressive races that have adapted to warmer climates have expanded the geographic footprint of this disease since 2000, resulting in severe losses in many countries (Wellings et al., 2012). Annual global losses due to stripe rust were recently estimated at USD $979 million (Beddow et al., 2015). Stripe rust is also considered the most damaging wheat rust disease in Australia, with annual economic losses valued at around AUD$127 million (Murray and Brennan, 2009). Stem rust is the most destructive wheat attacking rusts and has historically been especially damaging in Africa, the Americas, Europe, and Australia. Numerous severe stem rust epidemics occurred in the United States during the first half of the 20th century, causing average yield losses of 19.3 to 28.4% in some states (Roelfs, 1978). The wide utilization of resistant cultivars adopted during the Green Revolution and the eradication of the alternate host barberry since 1954 in the United States resulted in much improved global control of stem rust. However, this situation has changed following the emergence of the highly virulent stem rust pathotype Ug99, first detected in Uganda in 1998, and now widespread in parts of Africa and the Middle East (Singh et al., 2015).

Genetic control is considered as the most effective and environmentally friendly strategy to control rust disease and involves breeding effective disease resistance genes into wheat cultivars. Many rust resistance genes have been identified genetically, and introgression into wheat lines is increasingly being facilitated by the development of robust molecular markers. However, the massive and complex genome of wheat presents major challenges for the isolation of individual genes. In the past 17 years, 24 rust resistance genes have been cloned using various strategies, with more than half of these (15) identified only in the last 4 years. This recent accelerated progress was made possible by (i) the public availability of the first high quality reference genome for wheat (Chinese Spring RefSeq v1.0) and (ii) the development of various approaches for reducing the genome complexity to allow targeted resequencing analyses. In particular, 10 out of the 15 genes cloned since 2016 were identified through pipelines involving Target-sequence Enrichment and Sequencing (TEnSeq). In this review, we briefly covered some general features of the wheat-rust pathosystem and the most recent progress in the area of cloning wheat rust resistance and rust fungal effector genes.

## Rust Disease of Wheat

Rust fungi are one of the most diverse groups of plant pathogens, consisting of more than 120 genera and 6000 species (Duplessis et al., 2011). Studies of cereal rust diseases go back to Felice Fontana in 1767, who is considered to be the first person to provide a detailed description of cereal rusts and to recognize that rusts are caused by fungi (Fontana and Pirone, 1932; Chester, 1946). The primary causal agents of the wheat stem rust, leaf rust, and stripe rust diseases are *Puccinia graminis* Pers.: Pers. f. sp. *tritici* Erikss. & E. Henn (*Pgt*), *P. triticina* (syn. *P. recondita* Rob. ex Desm. f. sp. *tritici*) (*Pt*), and *P. striiformis* Westend. f. sp. *tritici* Erikss. & E. Henn. (*Pst*), respectively (Roelfs, 1985; Samborski, 1985; Stubbs, 1985; Knott, 1989; McIntosh et al., 1995). All belong to the genus *Puccinia*, family *Pucciniaceae*, order *Pucciniales*, class *Teliomycetes*, and phylum *Basidiomycota*, within the kingdom *Fungi*. Because of the economic importance of rust diseases, the causal agents are the most intensively studied plant pathogenic fungal species (McIntosh et al., 1995; Figueroa et al., 2020). Moreover, some important principles derived from the pathogenetic rust studies, for example, the gene-for-gene model (Flor, 1971), have found wide applications in other host–pathogen systems.

Rust pathogens are well-known to have great pathogenic variability, and the frequent emergence of new virulent strains that overcome resistance genes present in cultivated wheat varieties has hindered efforts to achieve durable resistance to these pathogens (Chen et al., 2014; Zhao et al., 2016). Studies of pathogenic variability in rust populations have indicated that new virulences can arise through the introduction of exotic genotypes, mutation in clonal lineages, sexual recombination and asexual hybridization (Park, 2007; Li et al., 2019). As an example, the *Pgt* population of Australia was postulated to be the result of four exotic incursions into the country since 1925 (Zwer et al., 1992; Zhang J. et al., 2017). Recent genetic studies have shown that three of these were derived from southern Africa and represent a single clonal lineage that was first described in the 1920s (Li et al., 2019; Visser et al., 2019). Subsequent to these introductions, stepwise mutations to overcome individual resistance genes have led to the divergence of numerous races with different pathotypes (Park, 2007).

## Genetic Resistance of Wheat Rust Disease

### Concept of Plant Innate Immunity

**FIGURE 1:**
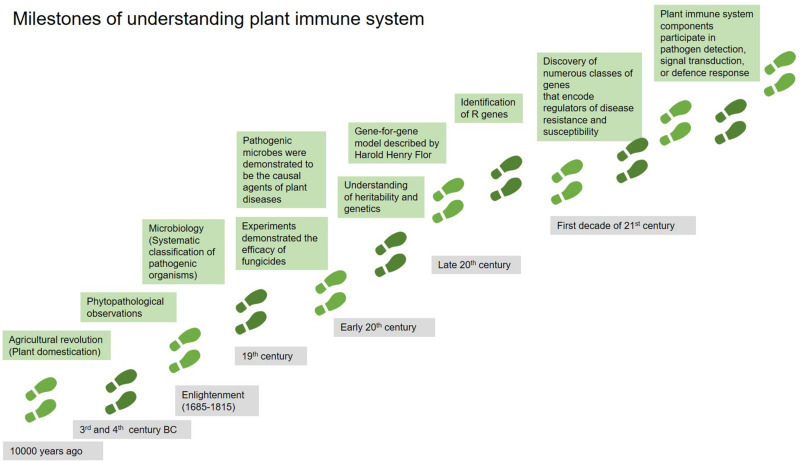
The milestones of understanding plant immune system in chronological order. Adapted from Andersen et al. (2018).

Long-term co-evolution between plants and their pathogens has equipped plants with a sophisticated multi-layered immune system to guard themselves against pest and pathogens (Andersen et al., 2018). The development of our understanding of the plant immune system is summarized in chronological order in Figure 1.

The plant immune system is often described in terms of two components, i.e., pattern-triggered immunity (PTI), activated by the recognition of microbial or pathogen-associated molecular patterns (MAMPs or PAMPs), and effector-triggered immunity (ETI), which encompasses “gene-for-gene” type of resistance (Jones and Dangl, 2006; Dodds and Rathjen, 2010). Bacterial flagellin and chitin are classic examples of MAMPs or PAMPs and are recognized by pattern recognition receptors (PRRs) such as Receptor-Like Kinase (RLK) and Receptor-Like Protein (RLP) type transmembrane receptor proteins. ETI is often based on the recognition of cytosolic effectors by immune receptors with a conserved nucleotide-binding domain (NB-ARC) and a leucine-rich repeat domain (LRR), hereafter referred to as NLRs. This type of resistance is normally associated with a hypersensitive response (HR) localized to infection sites. However, the distinction between PTI and ETI is often blurred, and recent models of plant immunity highlight that there are a range of cell-surface receptors recognizing extracellular ligands (which may be PAMPS or extracellular effectors or host derived-patterns), and intracellular receptors that recognize intracellular ligands (Cook et al., 2015; Kanyuka and Rudd, 2019).

### Plant Resistance (R) Genes

More than 300 R genes have been cloned from plants to date and Kourelis and van der Hoorn (2018) reviewed the defense mechanisms functions and summarized nine main defense mechanisms based on all cloned R proteins. Most plant R genes are dominant in action and encode immune receptors that recognize pathogen avirulence proteins as described above. However, some genes that confer resistance phenotypes operate via different mechanisms. For instance, *Hm1* from maize (*Zea mays*) was the first cloned R gene and encodes an enzyme that detoxifies a toxin from the fungal pathogen *Cochliobolus carbonum* (Johal and Briggs, 1992). Two wheat adult plant resistance (APR) genes *Lr34/Yr18/Sr57/Pm38* and *Lr67/Yr46/Sr55/Pm46* are also examples of non-immunity-mediated resistance genes (Krattinger et al., 2009; Moore et al., 2015). Both encode transporter proteins and confer resistance against multiple pathogens in wheat and can also function in other crops (Risk et al., 2013; Chauhan et al., 2015; Krattinger et al., 2016; Rinaldo et al., 2017; Schnippenkoetter et al., 2017; Sucher et al., 2018). These two genes have been important components of wheat breeding for rust resistance and often show additive or synergistic interactions with the other more “typical” R genes (immune receptors). Thus approaches for identifying effective resistance genes must consider both classical immune receptor class genes as well as other novel classes that may operate via different mechanisms.

### Plant NLRs

Most of the cloned R genes from the wheat-rust pathosystems encode immune receptors of the NLR class (19 out of 24, Table 1). Despite the huge evolutionary distance between the plant and animal kingdoms, members of both use the intracellular proteins of the NB-ARC-LRR superfamily to perceive pathogens. However, recent studies suggest that this is the result of convergent evolution and that the domain architecture of the NLRs evolved at least twice (Urbach and Ausubel, 2017). Jones et al. (2016) reviewed the processes associated with intracellular innate immunity in both plants and animals and built an NLR tree to illustrate the proposed evolution of NLR genes following independent pathways for plant and animal species. The authors also proposed that plant and animal NLRs evolved from two distinct derivatives of a common ancestral prokaryotic adenosine triphosphatase (ATPase) represented by the NB-ARC domain class (nucleotide-binding domain shared by APAF-1, plant R proteins, and CED-4) and the NACHT domain class (shared by NAIP, CIITA, HET-E, and TP1). While animal and fungal genomes can contain both NB-ARC and NACHT domains, no NACHT domains have been found in plants. NACHT domains are also absent from some animal taxa such as the nematodes and *Drosophila*.

**TABLE 1:** Cloned rust resistance genes in wheat from 2003 to 2020.

Gene	Type of protein encoded	Reference
*Lr21*	NLR	Huang et al., 2003
*Lr10*	NLR	Feuillet et al., 2003
*Lr1*	NLR	Cloutier et al., 2007
*Lr34/Yr18/Sr57/Pm38*	ABC transporter	Krattinger et al., 2009
*Yr36/WKS1*	Kinase-START	Fu et al., 2009
*Sr33*	NLR	Periyannan et al., 2013
*Sr35*	NLR	Saintenac et al., 2013
*Sr50*	NLR	Mago et al., 2015
*Lr67/Yr46/Sr55/Pm46*	Hexose transporter	Moore et al., 2015
*Sr22*	NLR	Steuernagel et al., 2016
*Sr45*	NLR	Steuernagel et al., 2016
*Lr22a*	NLR	Thind et al., 2017
*Sr13*	NLR	Zhang W. et al., 2017
*Sr21*	NLR	Chen et al., 2018
*Yr7*	NLR	Marchal et al., 2018
*Yr5* (*Yr5a*)	NLR	Marchal et al., 2018
*YrSP (Yr5b)*	NLR	Marchal et al., 2018
*Yr15*	Tandem kinase-pseudokinases	Klymiuk et al., 2018
*Sr46*	NLR	Arora et al., 2019
*SrTA1662*	NLR	Arora et al., 2019
*YrAS2388*	NLR	Zhang et al., 2019
*Sr60/WTK2*	Tandem kinase	Chen et al., 2019
*Sr26*	NLR	Zhang et al., under review
*Sr61*	NLR	Zhang et al., under review

The number of NLRs in a given plant genome can be as high as several 1000 (Appels et al., 2018). As revealed by the increasing number of newly available whole genome sequences and the more precise bioinformatic pipelines developed for identifying NLR genes, the number of NLR genes varies greatly between species. While the number of NLRs is normally proportional to the size of the genome, apple (*Malus domestica*) is an exception in possessing nearly 1,000 NLRs despite having a relatively small genome (740 Mb). In contrast, the number of NLRs in orchids species (*Apostasia shenzhenica*) with a genome size of 349 Mb was reported to be normally less than a 100 (Zhang G. Q. et al., 2017; Xue et al., 2020). This appears to indicate that the number of NLRs within a certain genome may also be a result of the selection pressures posed by pathogens during the evolutionary history of that plant lineage and degree of exposure to pathogens (Borrelli et al., 2018).

### The Wheat Genome and Its NLRs

The bread wheat (*Triticum aestivum*) genome is one of the most challenging plant genomes to study. It is highly repetitive (∼85%) and approximately 15.4–15.8 Gbp in size, which is five times larger than the human genome (Appels et al., 2018). *T. aestivum* is a hexaploid species that arose through natural hybridization of three closely related wild grass species which contributed the A, B, and D genomes of wheat (Salamini et al., 2002). A low coverage survey sequence of the wheat genome became available in 2012 (Brenchley et al., 2012), after which assemblies have been greatly improved as sequencing technologies and bioinformatic analysis pipelines became more powerful. The first version of the wheat genome in the form of chromosome-sized scaffolds (IWGSC RefSeq v1.0) was made publicly available in 2018 (Appels et al., 2018) and an improved version v2.0 in 2019. High-quality reference genome sequences were also recently published for the wild diploid progenitor of the wheat D genome (*Aegilops tauschii*) and the wild tetraploid progenitor *T. diccocoides* and cultivated tetraploid wheat *T. turgidum* cv. *durum* (Avni et al., 2017; Luo et al., 2017; Maccaferri et al., 2019). Based on an analysis of the IWGSC RefSeq v1.0 assembly Steuernagel et al. (2018) reported a total of 3,400 full-length NLR loci.

## TEnSeq Pipelines as a Catalyst in Isolating R Genes in Wheat

The massive and complex genome of wheat has made the isolation of individual genes a challenging task. Among ∼200 rust resistance genes cataloged in wheat, only a small number have been cloned and had their molecular functions studied. A complete list of the rust resistance genes cloned so far from wheat is shown in Table 1. In 17 years since 2003, there are in total 24 wheat rust R genes that have been cloned and published. More than half of the genes were cloned in the last 4 years after the MutRenSeq pipeline was published in 2016 (Steuernagel et al., 2016), and 10 of these were cloned directly or partially using approaches based on some version of genome complexity reduction which we refer to here collectively as TEnSeq (Target-sequence Enrichment and Sequencing).

**FIGURE 2:**
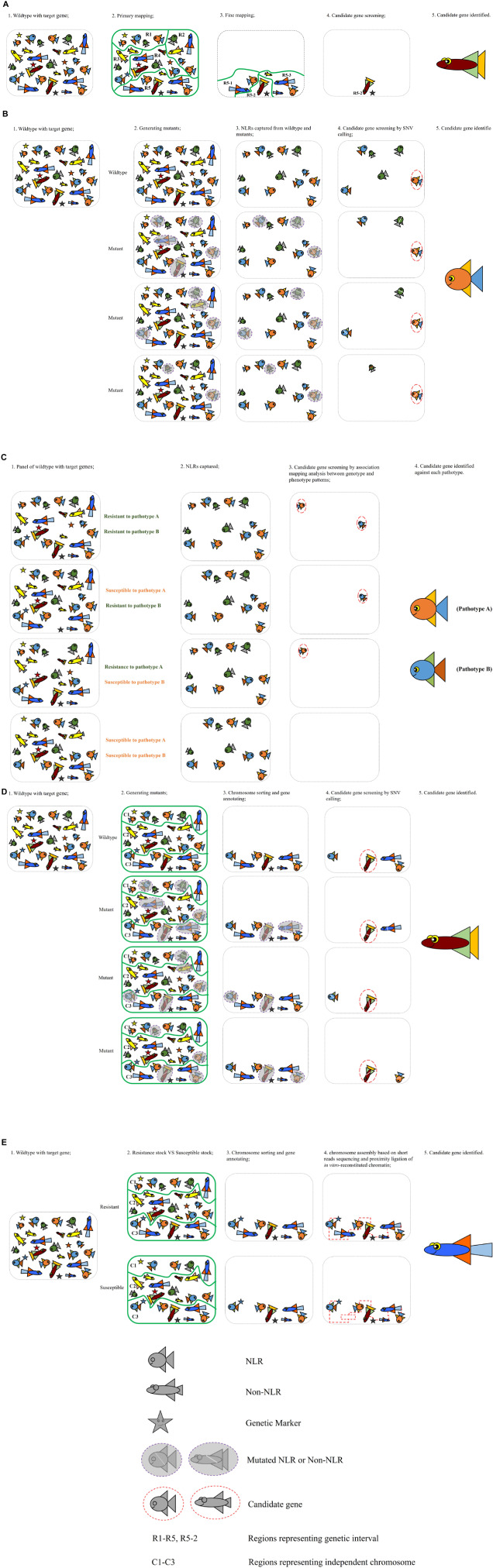
Illustrations of the five strategies applied for cloning resistance genes in wheat-rust pathosystem. **(A)** Map-based cloning; **(B)** MutRenSeq; **(C)** AgRenSeq; **(D)** MutChromSeq; **(E)** TACCA.

Various gene cloning strategies have been used to position a gene to its exact location within the genome and identify its nucleotide sequence and the protein that it encodes. The traditional map-based or positional cloning strategy narrows down the gene location by using genetic recombination in biparental populations that segregate for the gene of interest (Keller et al., 2018) (Figure 2A). This approach depends on the availability of a large segregating population that allows mapping of the gene of interest to a small genetic interval. Markers from this genetic interval are then used to screen a physical library, often prepared in bacterial artificial chromosomes (BACs). The large genome of wheat and relatively small size of the BAC clones (100–200 kb) requires that the target gene is mapped to a very small genetic interval that corresponds to only a few overlapping BAC clones. Thus this strategy is not viable for target genes derived from wild relatives of wheat and which are located in introgressed genome segments that do not recombine with wheat chromatin. Applying this strategy on genes that are located in centromeric regions is also extremely challenging, as recombination rates in this region are low. It is not uncommon to spend 5–10 years or even longer on cloning one gene by map-based cloning (Mago et al., 2015; Klymiuk et al., 2018).

To overcome the limitations of the map-based cloning strategy in the large genome of wheat, alternative approaches were developed and validated by the rapid cloning of several genes using TEnSeq pipelines. These include MutRenSeq (Mutagenesis and the Resistance gene Enrichment and Sequencing) (Figure 2B), AgRenSeq (Association genetics with R gene enrichment Sequencing) (Figure 2C), MutChromSeq (Mutgenesis Chromosome flow sorting and short-read Sequencing) (Figure 2D), and TACCA (Targeted Chromosome-based Cloning via long-range Assembly) (Figure 2E). The common component of these approaches is the intent to reduce the genome complexity prior to the use of next-generation sequencing (NGS). MutRenSeq and AgRenSeq are based on NLR-targeted DNA capture by hybridization, while MutChromSeq and TACCA rely on the purification of individual chromosomes from wheat lines. Comparisons of these newly developed approaches and the classical map-based cloning strategy are outlined in Table 2.

**TABLE 2:** Different strategies for cloning rust resistance genes in wheat.

Cloning strategies		Requirements	Advantages	Limitations	References	Wheat rust R gene cloned
Map-based cloning		High-resolution segregating mapping population	Generally suitable for cloning any types of genes, low cost	Laborious and time-consuming, difficult to apply on gene target situated in pericentromeric regions or from alien introgressions	Keller et al., 2005	*Lr1*, *Lr10*, *Lr21*, *Lr34/Yr18/Sr57/Pm38*, *Lr67/Yr46/Sr55/Pm46*, *Sr13*, *Sr21*, *Sr33*, *Sr35*, *Sr50*, *Sr60*, *Yr15*, *Yr36*, *YrAS2388*
Targeted-sequencs Enrichment and Sequencing (TEnSeq)	MutRenSeq	Loss-of-function mutants	Rapid cloning of NLR resistance gene from the large genome	Suitable only for cloning NLR-type resistance gene	Steuernagel et al., 2016	*Sr22*, *Sr26*, *Sr45*, *Sr61*, *Yr5*, *Yr7*, *YrSP*
	MutChromSeq	Loss-of-function mutants, chromosome flow sorting, chromosome location of the target gene	Rapid cloning of genes regardless of the type of the gene	Rely on chromosome isolation and the chromosome location of the target gene	Sanchez-Martin et al., 2016	N/A
	TACCA	Mapping population, chromosome flow sorting, long-range sequencing and assembly	Rapid cloning of genes from the large genome, regardless of the type of the gene	Rely on chromosome isolation. Requires map information of the gene	Thind et al., 2017	*Lr22a*
	AgRenSeq	Adequate diversity of pathotype for association analysis	Cloning NLR gene from diverse germplasm panel	Suitable only for cloning NLR-type resistance gene. Rely on the diversity of cognate pathotype	Arora et al., 2019	*Sr46*, *SrTA1662*

MutRenSeq was the earliest developed TEnSeq pipeline applied to clone rust resistance genes in the wheat genome. It is based on the Resistance gene Enrichment Sequencing (RenSeq) approach, which is an NLR gene-targeted, resistance gene enrichment and sequencing method. Jupe et al. (2013) described the RenSeq approach to improve the annotation of the NB-LRR gene repertoire from sequenced plant genomes, which are often poorly assembled due to the presence of complex gene families. As a proof of concept, they used it to reannotate and map NLRs in potato (*Solanum tuberosum*). The workflow was to first construct a customized target enrichment library (bait library) comprised of a series of 120-mer biotinylated RNA oligonucleotide. These were designed based on previously annotated NLR-like sequences from potato, tomato, tobacco, and pepper genomes and aimed to fully cover each NLR-gene sequence with bait probes of at least 80% sequence identity. This bait library was then used to capture and enrich the NB-LRR genes from a genomic DNA library of potato which was then sequenced using Illumina technology. Through this approach, the number of annotated NLRs in the potato genome was increased from 438 to 755. Furthermore, they applied RenSeq successfully in identifying SNP markers that co-segregate with the resistance against late blight pathogen *Phytophthora infestans* in two independent segregating populations of wild *Solanum* species.

Following the successful application of RenSeq on genetic mapping on the *Solanum* species, Steuernagel et al. (2016) proposed a three-step method, MutRenSeq, that combines mutagenesis with NLR gene capture and sequencing for rapid identification of resistance genes in wheat. Similarly, they designed a bait library containing 60,000 120-mer RNA probes with ≥ 95% similarity to predicted NLR genes present in the genome and transcriptome sequence data from Triticeae species including barley (*Hordeum vulgare*), hexaploid wheat (*T. aestivum*), tetraploid wheat (*T. durum*), red wild einkorn (*T. urartu*), domesticated einkorn (*T. monococcum*), and three goatgrass species (*Ae. tauschii*, *Ae. sharonensis*, and *Ae. speltoides*). The first successful application of MutRenSeq pipeline in cloning wheat rust resistance genes was the rapid cloning of wheat stem rust resistance genes *Sr22* and *Sr45*. Later, its high efficiency was again demonstrated through the successful and rapid identification of *Yr7*, *Yr5*, and *YrSP*, which are the first three cloned major R genes against wheat stripe rust (Marchal et al., 2018). The noticeable advantage of the MutRenSeq compared with the classical map-based cloning method is that it obviates the need for a high resolution segregating mapping family and for building a physical library contig that covers the genetic interval. This not only reduces the time involved in these processes but also makes it an ideal approach for targeting genes that are located in low recombination regions such as those derived from alien species. This application is demonstrated by the recent identification of the stem rust resistance genes *Sr26* and *Sr61*, which are located in a non-recombining introgressed segment from *Thinopyrum ponticum* (Zhang et al., under review).

The AgRenSeq pipeline also utilizes the NLR-gene capture method but integrates with Genome-Wide Association Studies (GWAS) to permit the cloning of R genes from a host diversity panel. Arora et al. (2019) validated this approach by identifying the wheat stem rust resistance genes *Sr46* and *SrTA1662* from a panel *Ae. tauschii* accessions. They also identified the previously cloned *Sr33* and *Sr45* genes within this diversity panel. In this approach, the GWAS analysis was based on the use of unique K-mer (sub-sequences) markers rather than single nucleotide polymorphisms. K-mers associated with resistance phenotypes were then used to identify the candidate NLRs.

MutChromSeq follows a similar basic principle to MutRenSeq of using multiple mutation events to identify candidate genes. However, the NLR gene-capture is replaced by whole chromosome isolation to reduce genome complexity. This approach requires that the chromosome location of the target gene is known so that the chromosome can be isolated from wild-type and mutant lines to allow sequence comparison. This approach circumvents one of the limitations of MutRenSeq, which is the requirement that a similar gene is represented in the bait library designed from existing pan-genome sequences. This approach was described first for cloning of the wheat powdery mildew resistance gene *Pm2* by Sanchez-Martin et al. (2016) and was later used on cloning the leaf rust resistance gene *Rph1* from barley by Dracatos et al. (2019). MutChromSeq also has the advantage that it does not rely on an underlying assumption that the resistance gene belongs to the NLR class, and therefore would be appropriate for identification of non-immune mediated resistance genes. Its most recent application is the cloning of Med15 encoded by *SuSr-D1*, a suppressor gene of stem rust resistance from the wheat cultivar ‘Canthatch’ (Hiebert et al., 2020).

The TACCA pipeline was first described by Thind et al. (2017) in the cloning of the leaf rust resistance gene *Lr22a*. It is essentially a map-based cloning strategy coupled with a cultivar specific chromosome assembly, which effectively increases the size of physical contigs onto which the genetic interval can be mapped. In this case, a sequence assembly was generated for chromosome 2D of a line carrying *Lr22a* after chromosome flow sorting and using a combination of Illumina short-read sequencing and proximity ligation of *in vitro*-reconstituted chromatin (Chicago long-range linkage). This allowed the identification of candidate genes within the physical interval delineated by the position of markers closely linked to *Lr22a*.

## Identifying Wheat Rust Effectors

Fungal effectors are proteins secreted by pathogens that facilitate infection, often by suppressing plant immunity to help the invasion of the host (Uhse and Djamei, 2018). Fungal effectors may act in the host cytoplasm or apoplast and are mostly represented by small secreted proteins. Although 300 amino acids in size is commonly adopted as a size cut-off for effector prediction, some exceptions to this limit exist, notably AvrSr35 from *Pgt*, which is about 600 aa. The prediction of fungal effectors was facilitated in recent years by the increasing availability of fungal genome sequence data, and especially the development of approaches to assemble the two haploid genomes of these dikaryotic organisms separately in the case of rust fungi (Miller et al., 2018; Schwessinger et al., 2018; Li et al., 2019). It also benefit significantly from the rapid development of machine learning-based effector prediction tools (Schwessinger et al., 2018). For rust fungi, most known avirulence proteins are cytosolic effectors that are delivered into host cells during infection from specialized haustoria structures and recognized by intracellular NLR-type resistance proteins (Garnica et al., 2014).

In the wheat-rust pathosystem two rust effector/Avr genes have been identified: namely *AvrSr50* and *AvrSr35* from *Pgt* (Chen et al., 2017; Salcedo et al., 2017), which are recognized by the corresponding *Sr50* and *Sr35* resistance genes in wheat. These were identified based on whole-genome sequencing of wild-type (avirulent) *Pgt* isolates and virulent mutants. These two *Pgt* effectors are quite distinct from each other in sequence, but both are Haustorial Secreted Proteins (HSPs). Coincidentally, the two genes are located adjacent to each other in the *Pgt* genome (Li et al., 2019). As is often the case for fungal effectors, these two *Avr* genes are unique to *P. graminis*, with no homologs in related rust species. Allele mining of *AvrSr50* and *AvrSr35* has identified numerous genetic variants associated with either virulence or avirulence phenotypes which can serve as predictors of *Pgt* pathogenicity on wheat lines carrying these resistance genes. An expanded repertoire of identified rust *Avr* genes will ultimately lead to the prediction of isolate virulence profiles from sequence data with applications in field-based molecular diagnostics.

## The Pursuit of Durable Resistance

### Resistance Gene Stewardship and Deployment

**FIGURE 3:**
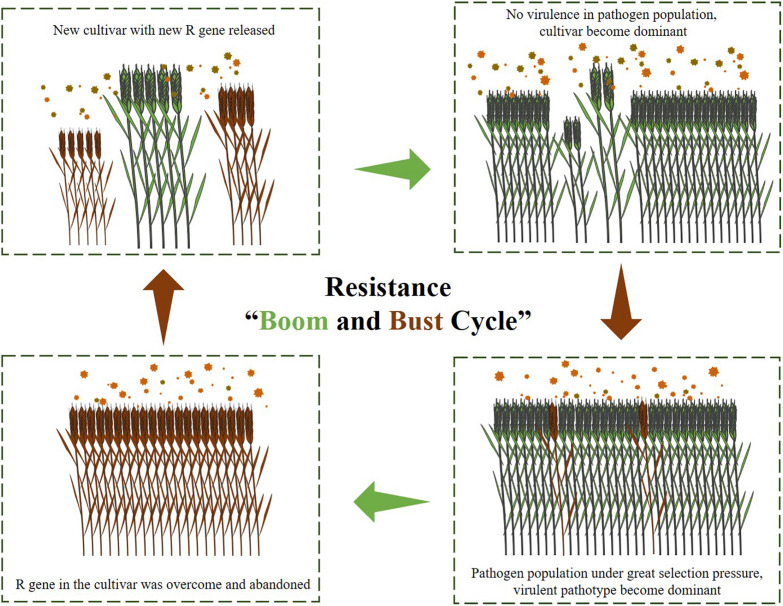
The illustration of the resistance “Boom and Bust Cycle.”

The increasing number of cloned wheat rust resistance genes in recent years has led to a reconsideration of how to deploy these newly cloned resistance genes in order to escape from the “boom and bust cycle” (Figure 3). Effective resistance gene stewardship refers to the careful and responsible management of resistance genes with the aim of prolonging the resistance effect (Pretorius et al., 2017). Gene pyramids are commonly considered as the best approach to gene stewardship as this minimizes the chance of the pathogen acquiring virulence through mutation. Backcrosses and transgenic gene cassettes are two practical methods for combining multiple resistance genes into the same background.

Effective resistance gene stewardship requires reference to the mechanism of resistance conferred by the available genes and their ability to work in combination. Rust resistance genes may be either race-specific or non-race-specific (Periyannan et al., 2017). Race-specific resistance refers to the resistance that is effective against some but not all races within a pathogen *formae specialis*, and generally follows the gene-for-gene model, e.g., the resistance occurs only when a specific immune receptor (R gene) encounters its corresponding effector (Avr gene). Most NLRs confers race-specific resistance and are effective in all growth stages of the host, therefore, these are often termed as all stage resistance (ASR). Race non-specific resistance describes resistance that is effective against all races of a pathogen species and sometimes may also be effective against multiple pathogens. These are normally quantitative traits conferring partial resistance that is able to slow down disease development. Wheat stem rust resistance gene *Sr2*, stripe rust resistance gene *Yr36*, and three multi-pathogen resistance loci *Lr34*, *Lr46*, and *Lr67*, all fall into this race non-specific category (Ellis et al., 2014). Most of the race non-specific resistance genes are effective only at the adult plant stage of the host and are therefore often described as adult plant resistance (APR) genes. The successful cloning of *Lr34*, *Yr36*, and *Lr67* since 2009 revealed these APRs encode an ABC transporter, a kinase-START protein, and a hexose transporter, respectively. They appear to each have their own resistance mechanism, function constitutively and often increase the basal level of resistance of the host, which is different from the recognition based NLRs. An important consideration is that some race-specific genes are also only effective at the adult plant stage, such as the NLR-encoding *Lr22a* (Thind et al., 2017).

Ellis et al. (2014) proposed that the most effective and durable means for genetic control of wheat rusts is the use of combinations of multiple broadly effective ASR and APR genes. Mundt (2018) in his latest review of durable resistance also suggested that resistance was likely to be more durable by pyramiding ASR genes into APR gene backgrounds. The APR genes *Sr2*, *Lr34*, and *Yr36* all have been reported to have some additive effects when in combination with certain seedling resistance genes. Given the distinct resistance mechanisms between APRs and ASRs based on current knowledge, these synergistic or additive resistance phenotypes between the APR and ASR are likely the result of combining two different modes of resistance.

In terms of gene stewardship, Pretorius et al. (2017) suggested that resistance genes could be classified into three groups with different management strategies required. R genes belonging to Group one do not need stewardship. This includes all the slow-rusting APR genes and the genes have already been extensively distributed in breeding programs and current wheat cultivars. Group two contains R genes that are publicly accessible but are yet to be deployed widely. Stewardship of these genes was strongly encouraged, especially by being deployed together with at least one other ASR gene that has broad resistance spectrum, or several APR genes. R genes in Group three refer to newly identified R genes that have never been deployed but potentially have high economic value. For this group, a minimum of three effective genes should be incorporated in order to withstand at least a double mutation to virulence in a pathogen. Patents or material transfer agreements could be employed to facilitate gene stewardship to act effectively, especially for R genes belonging to Group three. Pretorius et al. (2017) also indicated that stewardship is actually a result of the whole agricultural system, as the stewardship chain is only as strong as the weakest link. For example, an unintended release of a cultivar with a single resistance gene is enough to put this gene at risk of being overcome by the pathogen under strong selection pressure, no matter how much effort has been invested by the breeding community in generating gene pyramids containing this gene.

## Conclusion

There are roughly over 200 rust resistance genes that have been officially designated in wheat. Only a handful of these has been cloned to date, while many additional effective genes are expected to exist in wheat relatives. The advances in wheat rust resistance gene cloning reviewed in this paper, in particular, the TEnSeq pipeline which includes the MutRenSeq, MutChromSeq, TACCA, and AgRenSeq, will facilitate the identification of a much broader repertoire of wheat resistance genes. This will provide many more tools for marker-assisted selection in wheat breeding as well as the raw gene sequences to pursue gene stacking via transgenic gene cassettes. Together with advances in identifying genetic variation in rust *Avr* genes, these new tools should lead to more rational deployment strategies to maximize resistance durability. Although the TEnSeq strategies were initially developed specifically for resistance genes, these approaches can also be applied to genes with other functions and also adapted to other crops with large and complex genomes similar to wheat (Sanchez-Martin et al., 2016; Dracatos et al., 2019; Hiebert et al., 2020).

## Author Contributions

JZ wrote the manuscript. All authors reviewed and provided edits.

## Conflict of Interest

The authors declare that the research was conducted in the absence of any commercial or financial relationships that could be construed as a potential conflict of interest.
